# Differential spatial distribution of HNF4α isoforms during dysplastic progression of intraductal papillary mucinous neoplasms of the pancreas

**DOI:** 10.1038/s41598-023-47238-x

**Published:** 2023-11-16

**Authors:** Jahg Wong, Vincent Q. Trinh, Nidhi Jyotsana, Jumanah F. Baig, Frank Revetta, Chanjuan Shi, Anna L. Means, Kathleen E. DelGiorno, Marcus Tan

**Affiliations:** 1 Department of Pathology, University of Montreal, Montreal, QC Canada; 2 Institute for Research in Immunology and Cancer of the University of Montreal, Montreal, QC Canada; 3 Centre Hospitalier de l’Université de Montréal Research Center, Montreal, QC Canada; 4 Department of Surgery, Vanderbilt University Medical Center, Nashville, TN USA; 5 Cell and Developmental Biology, Vanderbilt University, Nashville, TN USA; 6 Department of Pathology, Microbiology and Immunology, Vanderbilt University Medical Center, Nashville, TN USA; 7 Department of Pathology, Duke University School of Medicine, Durham, NC USA; 8 Division of Surgical Oncology and Endocrine Surgery, Vanderbilt University Medical Center, 1211 Medical Center Drive, Nashville, TN 37232 USA; 9 Vanderbilt Ingram Cancer Center, Nashville, TN USA; 10 Vanderbilt Digestive Disease Research Center, Nashville, TN USA

**Keywords:** Pancreatic cancer, Pancreatic cancer, Oncogenesis

## Abstract

Hepatocyte Nuclear Factor 4-alpha (HNF4α) comprises a nuclear receptor superfamily of ligand-dependent transcription factors that yields twelve isoforms in humans, classified into promoters P1 or P2-associated groups with specific functions. Alterations in HNF4α isoforms have been associated with tumorigenesis. However, the distribution of its isoforms during progression from dysplasia to malignancy has not been studied, nor has it yet been studied in intraductal papillary mucinous neoplasms, where both malignant and pre-malignant forms are routinely clinically identified. We examined the expression patterns of pan-promoter, P1-specific, and P2-specific isoform groups in normal pancreatic components and IPMNs. Pan-promoter, P1 and P2 nuclear expression were weakly positive in normal pancreatic components. Nuclear expression for all isoform groups was increased in low-grade IPMN, high-grade IPMN, and well-differentiated invasive adenocarcinoma. Poorly differentiated invasive components in IPMNs showed loss of all forms of HNF4α. Pan-promoter, and P1-specific HNF4α expression showed shifts in subnuclear and sub-anatomical distribution in IPMN, whereas P2 expression was consistently nuclear. Tumor cells with high-grade dysplasia at the basal interface with the stroma showed reduced expression of P1, while P2 was equally expressed in both components. Additional functional studies are warranted to further explore the mechanisms underlying the spatial and differential distribution of HNF4α isoforms in IPMNs.

## Introduction

Intraductal papillary mucinous neoplasms (IPMNs) of the pancreas are pre-malignant cystic tumors of the pancreas, accounting for up to 25% of all cases of pancreatic ductal adenocarcinoma (PDAC)^1–3^. Unlike solid, non-IPMN PDAC and its precursor pancreatic intra-epithelial neoplasia (PanIN), IPMNs are easily identified by cross-sectional imaging (CT or MRI scans) and are typically diagnosed before invasive malignancy has developed. Thus, most patients with IPMN have a window of opportunity during which progression to cancer could be prevented., The mechanisms by which IPMN carcinogenesis occur remain obscure and are important to study, since PDAC has a dismal prognosis, and is projected to become the second leading cause of cancer-related death in the United States by 2030^4,5^.

HNF4α is a nuclear receptor superfamily of ligand-dependent transcription factors^6^. It is expressed in various visceral endodermal organs, and is considered a master regulator of hepatocellular differentiation with multiple roles in metabolic function and injury^6–10^. Alternative splicing of HNF4α’s two promoters, P1 and P2, yields up to twelve isoforms that are divided into P1 (α1-α6) and P2 (α7-α12) groups^11,12^. In the liver, mature hepatocytes predominantly express P1 isoforms which have metabolic, secretory and synthetic functions^9^. In contrast, hepatocytes that are immature, injured or dysplastic predominantly express P2 isoforms, which are associated with cellular dedifferentiation, proliferation and epithelial-to-mesenchymal transition (EMT)^9^. Beyond the liver, roles for HNF4α have been identified in several malignant and pre-malignant lesions including Barret’s esophagus, gastric intestinal metaplasia, gastric adenocarcinoma, colorectal carcinoma and PDAC^13,14^. In these, HNF4α dysregulation is associated with alterations in transcription networks related to metabolic, inflammatory and proliferative pathways^7,14–16^. Transcriptomic studies of non-IPMN PDAC have described “classical” and “basal/squamous” molecular subtypes^17–20^. These studies have shown HNF4α predominantly in the classical molecular group of PDAC while loss of HNF4α is associated with the basal subtype^21^. Additional studies employing cell lines, mouse models, and human tissue have studied HNF4α in PDAC but not in IPMNs^21,22^.

Specific expression patterns of P1 and P2 isoform groups vary between cancers of different anatomical origins^13,14,23,24^. There is also subcellular and spatial variation in HNF4α isoform expression, such as colonic mucosa where P1 isoforms are expressed in the differentiated surface component whereas P2 expression is predominant in the proliferative basal crypt cells. The nuclear localization of P1, however, is lost or shifted to the cytoplasm with progression to colorectal carcinoma^25^. However, the subcellular and spatial distribution of HNF4α remains poorly studied in the normal and neoplastic pancreas.

HNF4α’s potential role in tumorigenesis makes it a target of interest for potential therapeutic interventions. For instance, restoration of HNF4α delivered through lipid nanoparticles in human fibrotic liver tissue has been shown to attenuate fibrosis and cirrhosis^26^. Similarly, ectopic HNF4α expression in hepatocellular carcinoma (HCC) is associated with increased miR-122 expression, which induces re-differentiation, mesenchymal-to-epithelial transition, and decreased invasive capacity^27^. HNF4α’s role in PDAC represents an opportunity to translate findings in HCC to pancreatic neoplasms such as IPMNs^21^.

In the present study, we have explored HNF4α expression in IPMNs using promoter-specific antibodies. We show that nuclear HNF4α expression increases in high-grade dysplasia and in well-differentiated invasive IPMN but is lost in poorly differentiated invasive IPMN. Additionally, we show that subcellular and spatial distribution of HNF4α expression varies in pan-promoter and P1 isoform but not for P2 isoform groups. Our study serves as the basis for future functional studies that can further characterize the roles of specific HNF4α isoforms in IPMN carcinogenesis. Further understanding of mechanisms underlying IPMN development may provide avenues for the development of novel therapies.

## Materials and methods

### IPMN selection

This study was approved by the Vanderbilt University Human Research Protections Program (Protocol #101,066; Nashville, TN). The need for written informed patient consent was waived by Vanderbilt University (Vanderbilt University Human Research Protections Program; Protocol #101,066; Nashville, TN). HIPAA identifiers were deleted to assure data anonymity. This study was conducted in accordance with the Declaration of Helsinki. After IRB approval, surgical specimens of human IPMN were retrospectively selected from institutional and referred patients to Vanderbilt University Medical Center (VUMC, Nashville, TN, USA). Morphologically, IPMNs were of either gastric-foveolar (GF), intestinal (INT), or pancreaticobiliary (PB) subtype (Table 1). Non-invasive IPMNs were also categorized as low-grade (LG) or high-grade (HG). Invasive glandular components associated with IPMN were well-differentiated if they exhibited well-defined glandular architecture or poorly differentiated if glandular architecture was lost. In clinical practice, grading of invasive PDAC is determined by the proportion of tumor composed of well-formed glands. In practice, PDAC is heterogenous, and may often be “moderately differentiated” when 50–95% of the total tumor consists of glands^28^. To capture the heterogenous intra-tumoral morphology of PDAC, expression patterns were recorded based on morphology of individual well-differentiated and poorly differentiated components, rather than on final tumor grade that simply represents the dominant degree of glandular differentiation. Controls consisted of normal pancreatic or duodenal tissue without IPMN.

**Table 1:** Total cohort IPMN cases by morphological subtype, and grade of dysplasia.

	GF	INT	PB	Total
Low grade IPMN	16	7	5	28
High grade IPMN	5	4	9	18
Invasive	1	5	3	9
Total	22	16	17	55

*GF*: Gastric-foveolar; *INT*: Intestinal, *PB*: Pancreaticobiliary; *IPMN*: Intraductal papillary mucinous neoplasm.

### Immunohistochemistry (IHC) testing

Tissue sections were stained by H&E, according to standard protocol for diagnostic purposes. Monoplex immunohistochemistry was then conducted using a standard protocol with sodium citrate pH6.0 heat-induced epitope retrieval with DAB (K3468; Dako), and counterstained with Mayer’s hematoxylin (S3309; Dako). For each of the 55 specimens, one slide per specimen was chosen for immunohistochemical testing with a rabbit monoclonal antibody directed against pan-promoter HNF4α isoforms, which includes both P1 and P2-specific isoform groups (EPR3648, ab92378). A second cohort comprising 31 specimens with 60% diagnosed as low-grade IPMN and 40% with high-grade or invasive IPMNs underwent additional immunohistochemistry with antibodies specific to P1 isoform-specific HNF-4-alpha antibodies (K9218, PP- PP-K9218-00, 2ZK9218H, R&D Systems) and P2 isoform-specific HNF-4-alpha/NR2A1 antibodies (H6939, PP-H6939-00, R&D Systems). HNF4α immunohistochemical staining was considered satisfactory if the pathologically reviewed elements showed at least weak/focal positivity. This was not the case for 2/31 (6%) specimens stained with P2-isoform-specific HNF-4-alpha/NR2A1 antibody that were excluded from the study. A SCN400 slide scanner (Leica, Wetlzar, Hesse, Germany) was used to scan whole slides at 20X objective magnification. Normal human pancreatic and duodenal tissue were used as controls.

### Pathology validation and analysis

All cases were independently reviewed by two pathologists, one of whom was specialized in gastrointestinal and pancreaticobiliary pathology. QuPath version 0.3.2 was used to visualize and annotate scanned whole slide images^29^. Interobserver discordance resulted in a review by both observers to establish consensus. Surgical specimens were reviewed to confirm morphological subtype, grade of dysplasia, and grade of invasive components.

For each slide, up to 3 regions of interest measuring 250 × 250 µm^2^ were selected to evaluate each of the following components: pancreatic acini, intercalated ducts, intralobular ducts, large ducts, peribiliary glands, acinar-to-ductal metaplasia (ADM), surface low-grade IPMN components, basal low-grade IPMN components, surface high-grade IPMN components, basal high-grade IPMN components, well-differentiated invasive adenocarcinoma arising from IPMN, and poorly differentiated invasive adenocarcinoma component arising from IPMN (Table 2). Surface IPMN components were defined as epithelial cells lining fibrovascular papillary projections. Basal IPMN components were defined as epithelial cells in contact with non-papillary stroma. Certain regions of interest were used to evaluate more than one component. The QuPath interactive alignment function was used select the same region of interest (ROI) in serial sections of a given tissue.

**Table 2:** Number of regions of interest scored per component and HNF4α isoform.

Component	Isoform		
	Pan-promoter HNF4α (*n* = 55)	HNF4α P1 (*n* = 31)	HNF4α P2 (*n* = 29)
Acini	84	39	39
Intercalated ducts	84	39	39
Intralobular ducts	84	38	38
Large ducts	42	21	22
Peribiliary glands	39	20	19
ADM	69	32	33
Low-grade IPMN, surface	105	58	58
Low-grade IPMN, base	109	62	62
Low-grade IPMN, total	214	120	120
High-grade IPMN, surface	81	42	46
High-grade IPMN, base	76	36	38
High-grade IPMN, total	157	78	84
Well-diff INV	24	12	12
Poorly diff INV	15	6	6

*ADM*: Acinar-to-ductal metaplasia; *IPMN*: Intraductal papillary mucinous neoplasm; Well diff *INV*: Well-differentiated invasive adenocarcinoma; Poorly diff *INV*: Poorly differentiated invasive adenocarcinoma.

For each region of interest targeting a specific component, HNF4α IHC expression was graded between 0 and 3: “0” represented absent expression; “1” represented areas in which under 50% of epithelial cells showed up to moderate staining; “2” represented areas in which over 50% of epithelial cells exhibited moderate staining but under 50% of cells showed strong staining; “3” represented areas with a strongly diffuse staining in over 50% of epithelial cells.

### Statistical analyses

Different set sizes were used for different statistical analyses due to logistical restraints. In statistical tests comparing HNF4α expression in non-invasive IPMN without regard for spatial distribution, the scored regions of interest at the surface and at the base were combined. In statistical tests comparing the difference in HNF4α expression between the surface and basal components of non-invasive IPMN, the corresponding regions of interest were separately compared. Mann–Whitney U with two-tailed significance level of 0.05 and Kruskal Wallis non-parametric testing were conducted using GraphPad Prism version 8.4.3 (GraphPad Software, San Diego, CA, USA).

## Results

### Nuclear HNF4α expression increases in normal ductal components

First, we sought to characterize nuclear pan-HNF4α expression patterns in normal pancreatic tissue (Supplemental Fig. S1). Expression of HNF4α in acini was weak and focal. As intercalated ducts progressed to intralobular ducts and then larger pancreatic ducts, the intensity of nuclear pan-HNF4α expression increased. (Kruskal–Wallis P < 0.0001, Supplemental Fig. S1B). We observed an increased expression of pan-HNF4α in ADM, consistent with previous studies showing increased HNF4α expression as acini and benign ducts undergo an ADM-PanIN-PDAC sequence of progression^21,22^. A similar pattern of higher expression in larger ducts was observed for the HNF4α P1 isoform group (Kruskal–Wallis *p* < 0.0001, Supplemental Fig. S2) and the HNF4α P2 isoform group (Kruskal–Wallis *p* < 0.0001, Supplemental Fig. S3).

### Nuclear HNF4α expression increases with IPMN grade

**Figure 1:**
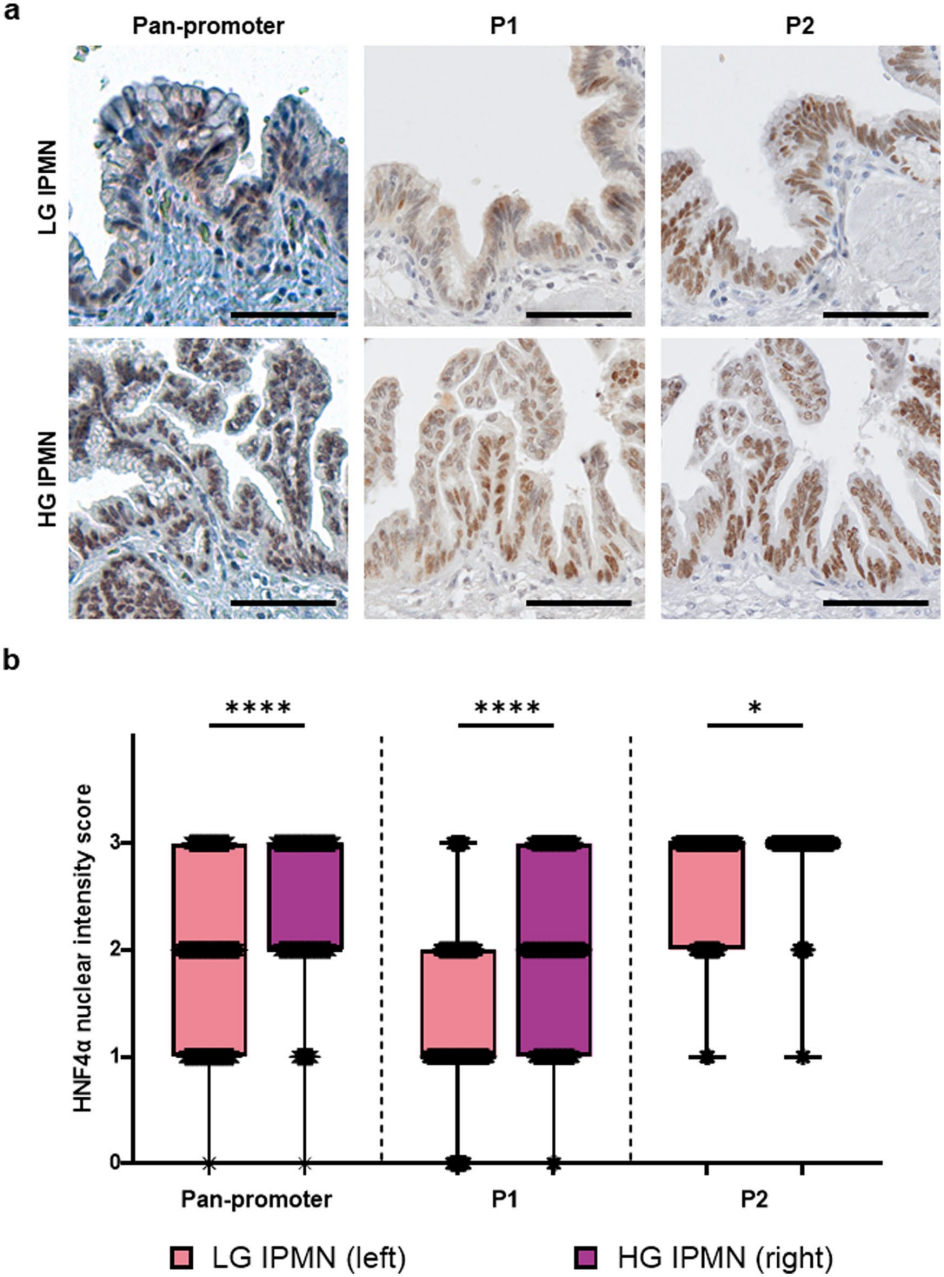
Increased nuclear expression of HNF4α isoforms in non-invasive HG IPMNs. (**a**) Average nuclear staining intensity of non-invasive IPMN ROIs as graded by 2 pathologists for pan-promoter, P1, and P2 HNF4α isoforms. The scale bar represents 100 µm. (**b**) Average nuclear intensity according to IPMN grade. Pan-promoter LG IPMN versus HG IPMN: *p* < 0.0001; P1 LG IPMN versus HG IPMN *p* < 0.0001; P2 LG IPMN versus HG IPMN: *p* = 0.0211.

For non-invasive IPMN, we observed a higher nuclear expression of HNF4α in high-grade IPMN relative to low-grade IPMN (Fig. 1a,b). This increase was significant in pan-promoter (*p* < 0.0001), P1 (*p* < 0.0001, and P2 (*p* = 0.0211) isoform groups of HNF4α. The increased nuclear expression of HNF4α in non-invasive IPMN mirrors the expression pattern observed in PanIN in which isoform-specific expression patterns have not been studied, and in intestinal metaplasia in the stomach, which is characterized by synchronously increased P1 and P2 isoform expression^14,15,21,22^.

### Nuclear HNF4α expression decreases between well-differentiated and poorly differentiated invasive components

**Figure 2:**
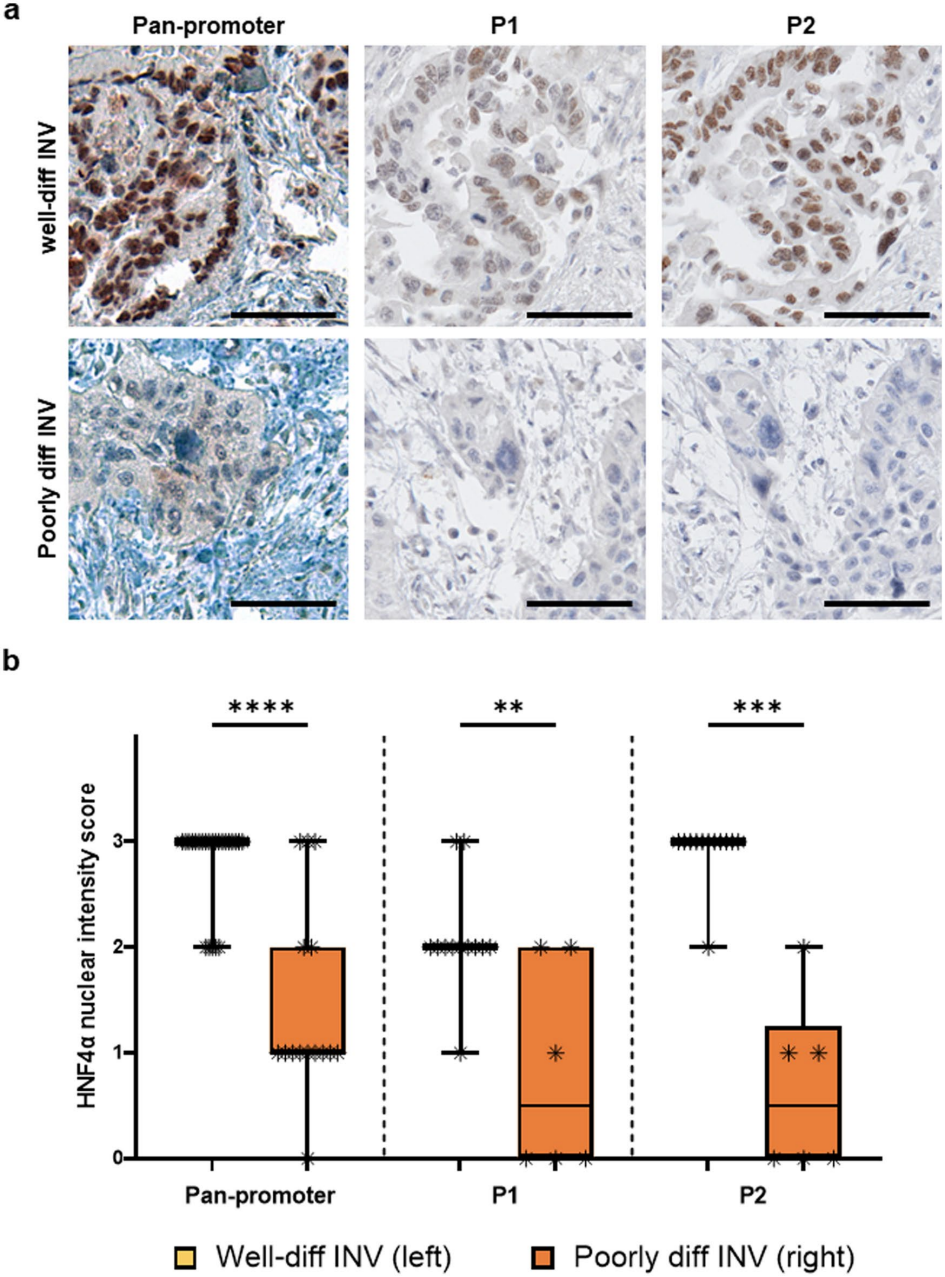
Loss of HNF4α nuclear expression isoforms in poorly differentiated invasive IPMN. (**a**) Average nuclear staining intensity of invasive carcinoma ROIs associated with IPMNs, as graded by 2 pathologists for pan-promoter, P1, and P2 HNF4α isoforms. The scale bar represents 100 µm. (**b**) Average nuclear intensity according to differentiation of invasive carcinoma component. Pan-promoter well-diff INV versus poorly diff INV: *p* < 0.0001; P1 well-diff INV versus poorly diff INV: *p* = 0.0066; P2 well-diff INV versus poorly diff INV: *p* = 0.0001.

Next, we evaluated HNF4α expression patterns in well-differentiated and poorly differentiated components of invasive adenocarcinoma associated with IPMN. Pan-promoter HNF4α nuclear expression was higher in well-differentiated invasive IPMN compared to HG non-invasive IPMN (*p* = 0.0026; Supplemental Fig. S1) but this difference was not observed for P1-specific (*p* = 0.8021; Supplemental Fig. S2) and P2-specific isoform groups (*p* = 0.6260; Supplemental Fig, S3). However, HNF4α nuclear expression was markedly lower in poorly differentiated invasive IPMN compared to well-differentiated invasive IPMN across all isoform groups: pan-promoter (*p* < 0.0001), P1 (*p* = 0.0066), and P2 (*p* = 0.0001) (Fig. 2a,b). The loss of HNF4α in poorly differentiated invasive IPMN components mirrors expression patterns observed in PDAC^21,22^. Similarly, HNF4α nuclear expression in poorly differentiated invasive IPMN was lower than in high-grade non-invasive IPMN across all isoform groups: pan-promoter (*p* = 0.0002), P1 (*p* = 0.0087), and P2 (*p* < 0.0001). Poorly differentiated invasive components showed a lower HNF4α nuclear expression than low-grade non-invasive IPMN for pan-promoter (*p* = 0.0384) and P2 (*p* < 0.0001) isoform groups but not for the P1 isoform group (*p* = 0.1808) (Supplemental Fig. S1, S2, S3).

### Variations in subcellular localization of HNF4α expression are observed for pan-promoter and P1 but not P2 isoform groups

**Figure 3:**
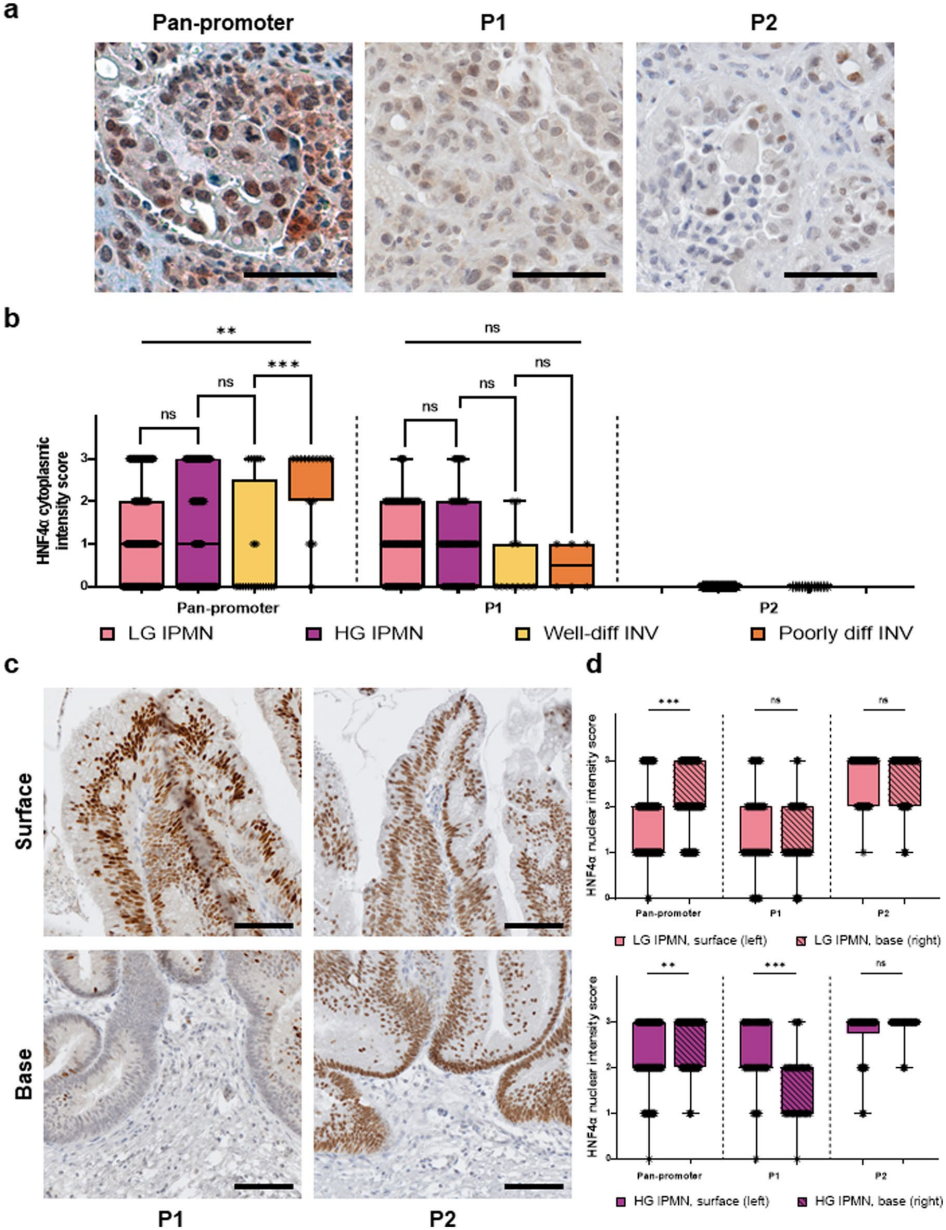
Differential staining of P1 and P2 in tumor cell components. (**a**) Average cytoplasmic staining intensity of IPMN and invasive carcinoma ROIs, as graded by 2 pathologists for pan-promoter, P1, and P2 HNF4α isoforms The scale bar represents 100 µm. (**b**) Pan-promoter LG IPMN versus HG IPMN : *p* = 0.9186; Pan-promoter HG IPMN versus well-diff INV: *p* = 0.1108; Pan-promoter well-diff INV versus poorly diff INV: *p* = 0.0005; Pan-promoter Kruskal–Wallis: *p* = 0.0014; P1 LG IPMN versus HG IPMN: *p* = 0.5553; P1 HG IPMN versus well-diff INV: *p* = 0.0519; P1 well-diff INV versus poorly diff INV : *p* = 0.8045; P1 Kruskal–Wallis: *p* = 0.0722; No cytoplasmic staining was observed for P2 isoforms. (**c**) Differential staining of P1 and P2 in surface versus base of high grade IPMN ROIs. The scale bar represents 250 µm. (**d**) Pan-promoter low grade IPMN, surface versus base: *p* = 0.0009; P1 low grade IPMN, surface versus base: *p* = 0.3634; P2 low grade IPMN, surface versus base: *p* = 0.6862; Pan-promoter high grade IPMN surface versus base: *p* = 0.0049; P1 high grade IPMN, surface versus base: *p* = 0.0001; P2 high grade IPMN, surface versus base : *p* = 0.0567.

We then sought to characterize the cytoplasmic expression of HNF4α isoform groups in IPMN and invasive components. In the pan-promoter HNF4α isoform group, poorly differentiated invasive components showed an increase in cytoplasmic expression relative to well-differentiated invasive components (*p* = 0.0005), but there was no difference in cytoplasmic pan-promoter HNF4α expression between low-grade and high-grade IPMN components (*p* = 0.9186) or between high-grade and well-differentiated invasive components (*p* = 0.1108). Cytoplasmic expression was observed within the P1 isoform group, though there was no statistically significant difference among non-invasive or invasive IPMN components (Kruskal–Wallis *p* = 0.0722). In contrast to pan-promoter and P1 isoform groups that showed both nuclear and cytoplasmic HNF4α expression, P2 expression was strictly nuclear in non-invasive IPMN and invasive components (Fig. 3a,b).

### Variations in spatial HNF4α distribution are observed for pan-promoter and P1 but not P2 isoform groups

Additionally, we compared nuclear HNF4α expression between surface and basal epithelium of non-invasive IPMN. There was increased pan-promoter HNF4α expression in the basal compartments of low-grade IPMN (*p* = 0.0009) compared to the surface compartment, but this pattern was not observed in P1 or P2 isoform groups. In high-grade non-invasive IPMN, pan-promoter HNF4α was increased in the basal compartment (*p* = 0.0049) but the surface IPMN epithelium exhibited increased P1 isoform-specific expression relative to basally located cells at the stromal interface (*p* = 0.0001). There was no significant difference between surface and basal expression for P2 isoforms in either low-grade or high-grade IPMN components (Fig. 3c,d).

## Discussion

Our results demonstrate that HNF4α expression may be dynamically involved in IPMN dysplastic progression and invasive transformation. We showed that there is pan-promoter increase in HNF4α expression through IPMN dysplasia and in well-differentiated invasive components. There is however a loss of HNF4α expression in poorly differentiated components. In addition, we showed that P1 isoform groups show variable subcellular localization and there is also variable spatial distribution of P1-specific isoforms in high-grade disease. In contrast, P2 isoform expression is constant throughout IPMN progression except for poorly differentiated invasive lesions that lost expression. Our findings complement the literature on HNF4α in PanIN-associated PDAC, while characterizing HNF4α expression patterns for the first time in IPMN^15,21,22^.

There are conflicting results pertaining to the HNF4α isoform-specific expression in non-tumoral pancreatic tissue. Human pancreatic tissue stained by Tanaka et al. exclusively expressed pan-promoter and P2 isoforms, while P1 isoforms were absent^13^. Conversely, a transcriptional study by Eeckhoute et al. showed the presence of P1 isoforms and absence of P2 isoforms in human exocrine pancreas cell lines^30^. In addition, Camolotto et al. observed patient-derived xenograft models of PDAC to show expression of either P2 only or concomitant P2 and P1 isoform group expression^21^. Our findings were nonetheless consistent with previous studies, since the nuclear intensity score was generally higher for P2 than P1 in normal exocrine components.

Variations of HNF4α expression patterns in different cancer types reflect the protean functions of HNF4α isoform groups^31,32^. The isoform-specific expression pattern we observed in IPMNs mirrors the expression patterns of certain gastrointestinal tract adenocarcinomas such as esophageal, gastric, and pancreatic adenocarcinoma^31,33^. These tumors all involve pre-neoplastic metaplastic processes, such as gastric intestinal metaplasia, Barret’s esophagus, and pancreatic ADM which are also similarly associated with increased overall HNF4α expression^14,15,21,22,34^. When looking at the isoform-specific differences in these metaplastic processes, gastric intestinal metaplasia is associated with P1-specific overexpression in a background of P2 expression within normal foveolar cells, while isoform-specific expression has not yet been well characterized in Barret’s esophagus and ADM^15^. This overexpression pattern may reflect a rerouting of P1-mediated cell differentiation towards other tumor-promoting mechanisms^31,35–37^. In contrast, hepatocellular carcinoma is characterized by decreased expression of P1 isoforms. Acute or chronic cellular injury resulted in decreased HNF4α loss, due to inflammatory pathways such as NF-κB that induce endoplasmic reticulum stress and impair HNF4α recruitment for normal metabolic functions, as observed in models of chronic hepatocyte injury, cirrhosis, and colitis^9,16^. In these specific contexts, it is possible that P1 isoforms instead have a tumor suppressive function in which sustained loss leads to metabolic reprogramming and HCC. The loss of pan-isoform HNF4α expression in poorly differentiated invasive IPMN may reflect a severe loss of HNF4α-dependent cell differentiation due to accumulated epigenetic and genetic alterations. The loss of pan-isoform HNF4α we observed in poorly differentiated invasive IPMN reflects prior observations in PanIN-associated PDAC. In human PDAC cell lines, Kim, et al. showed that HNF4α had increased expression in PanIN and in well-differentiated PDAC, but was lost in undifferentiated carcinoma^22^. Similarly, Camolotto et al. used murine and human models to show that HNF4α deletion resulted in poorly differentiated PanIN-derived PDAC, and that reconstitution of HNF4α isoform 8, a P2 isoform, resulted in decreased tumoral proliferation and increased epithelial differentiation^21^. Modulation of tumor differentiation may be attributed to HNF4α-mediated inhibition of mesodermal lineage markers SIX1 and SIX4 that are activated in the basal molecular subtype of PDAC^21^. Further mechanistic studies are required to establish the exact biological roles of individual HNF4α isoforms in IPMN progression.

The isoform-specific subcellular localization of HNF4α may be related to post-translational and epigenetic factors that differentially modulate HNF4α expression. In colon adenocarcinoma, P1 isoform expression that is normally present in the superficial differentiated epithelium is lost and instead exhibits a cytoplasmic shift due to SRC-mediated phosphorylation and degradation of HNF4α^25^. Phosphorylation-mediated cytoplasmic HNF4α retention has also been described in mice with hepatic steatosis^8^. Other processes that may interfere with HNF4α transcriptional activity include epigenetic methylation which is observed in hepatocyte injury and in the squamous/basal molecular subtypes of PDAC^9,38^.

Variability in the spatial distribution of HNF4α isoforms may similarly reflect variable isoform functions. The micro-anatomical distribution of HNF4α has previously only been studied in human and murine colon tissue in which the differentiated surface component is characterized by P1 predominance while the proliferative basal compartment showed a P2 predominance. In humans, HNF4α P1 isoform is downregulated in colorectal carcinoma at the transcriptional and proteinic level by WNT/β-catenin activity, whereas P2 isoforms are maintained throughout tumorigenesis^39^. Restriction to α1 or α7 expression in a model of colitis resulted in concomitant surface and basal expression of the restricted isoform, though colorectal carcinoma with α1 isoform restriction showed lower tumor burden compared to α7 restriction^16^. In high-grade IPMN, we observed an increase in surface expression of P1 relative to the basal compartment. This increase in surface P1 expression suggests reprogramming of surface cells before eventual progression to invasive carcinoma. In contrast, the lower degree of P1 expression within the basal compartments in association with an increase in P2 is more similar to the pattern observed in colonic crypts with proliferative capacity. This differential expression between the surface and the base may potentially be explained by the differences in stromal signaling that modulate HNF4α expression. For instance, in the liver, increased extracellular matrix rigidity secondary to liver fibrosis activates YAP nuclear translocation and results in HNF4α downregulation^40^. Similarly to colorectal carcinoma, a subset of PDACs with low HNF4α expression are characterized by an amplified WNT/β-catenin signaling program and an increased tolerance to GSK3β inhibitors^38^. The potential role of the IPMN tumor-microenvironment in HNF4α modulation has yet to be studied.

The limits of our study are mainly attributable its retrospective methodology. Although our study shows an association of HNF4α immunohistochemical expression patterns within IPMN progression, a causal relationship cannot be determined. However, the increased HNF4α expression in high-grade dysplasia and well-differentiated invasive adenocarcinoma, with subsequent loss of expression in poorly differentiated invasive adenocarcinoma, is a common event observed for pan-promoter, P1 and P2 isoforms. Although the employed semi-quantitative scoring for immunohistochemical expression of HNF4α is subject inter-observer variability, our results examined the relative differences in HNF4α immunohistochemical expression scores, rather than the absolute scores. In vitro study of HNF4α in IPMN is hampered by lack of cell lines and mouse models. Fresh tissue is only available at the time of surgical resection, but collection for research is often impossible when the entire specimen must be submitted to assess the presence of an invasive component. Furthermore, the antibodies employed for immunohistochemistry target pan-promoter, P1 and P2 isoform groups without further discrimination of the underlying isoforms within these groups. When comparing surface to basal HNF4α expression, we observed discrepant patterns between pan-promoter, P1 and P2-specific isoform expression. For instance, low-grade IPMN showed increased basal pan-promoter expression relative to surface expression, yet this was not observed with P1 or P2-specific antibodies. High-grade IPMN similarly showed a higher degree of basal pan-promoter specific expression relative to surface epithelium but P1 isoforms showed increased surface expression relative to the basal epithelium while P2 expression did not vary. These discrepant expression patterns may be due to post-translational HNF4α protein modifications, such as phosphorylation and acetylation, that may variably affect protein stability and epitope recognition by immunohistochemical antibodies^25,41^.

Our study serves as a basis for future studies that will delve into the roles and differential distributions and functions of specific isoforms, particularly in high-grade disease. Prevailing single-cell sequencing analysis studies have revealed a highly heterogenous and complex genetic landscape in pancreatic adenocarcinoma and its precursors, which have been shown to also harbor a wide array of metaplastic programs as well as a heterogenous tumor microenvironment^42–46^. While many of these reports analyze pan-gene transcripts, the relevance of HNF4α isoform expression in addition to subcellular and spatial distribution, adds another layer of complexity to our understanding of IPMN carcinogenesis.

In summary, we showed that nuclear HNF4α expression is increased in IPMNs with high-grade dysplasia and well-differentiated invasive IPMN but is lost in poorly differentiated invasive IPMN. We also demonstrated that pan-promoter and P1-specific isoforms show variable cytoplasmic expression of HNF4α. Similarly, there is a differential spatial distribution of P1 isoforms in high-grade IPMNs. These findings can serve as a basis for investigating the roles specific HNF4α isoforms in IPMN progression, as well as their viability as potential therapeutic targets.

### Supplementary Information

Supplementary Legends.

Supplementary Figure S1.

Supplementary Figure S2.

Supplementary Figure S3.

## Data Availability

All data generated or analysed during this study are included in this published article (and its Supplementary Information files).

## References

[1] Maitra A, Fukushima N, Takaori K, Hruban RH (2005). Precursors to invasive pancreatic cancer. Adv. Anat. Pathol..

[2] Muraki T (2022). Pancreatic ductal adenocarcinomas associated with intraductal papillary mucinous neoplasms (IPMNs) versus pseudo-IPMNs: relative frequency, clinicopathologic characteristics and differential diagnosis. Mod. Pathol..

[3] Hu Y (2018). Comparative effectiveness of resection vs surveillance for pancreatic branch duct intraductal papillary mucinous neoplasms with worrisome features. JAMA Surg..

[4] Rahib L (2014). Projecting cancer incidence and deaths to 2030: the unexpected burden of thyroid, liver, and pancreas cancers in the United States. Cancer Res..

[5] Siegel R, Ma J, Zou Z, Jemal A (2014). Cancer statistics, 2014. CA Cancer J. Clin..

[6] Walesky C, Apte U (2015). Role of hepatocyte nuclear factor 4alpha (HNF4alpha) in cell proliferation and cancer. Gene Expr..

[7] Yeh MM, Bosch DE, Daoud SS (2019). Role of hepatocyte nuclear factor 4-alpha in gastrointestinal and liver diseases. World J. Gastroenterol..

[8] Yu D (2018). High fat diet-induced oxidative stress blocks hepatocyte nuclear factor 4alpha and leads to hepatic steatosis in mice. J. Cell Physiol..

[9] Argemi J (2019). Defective HNF4alpha-dependent gene expression as a driver of hepatocellular failure in alcoholic hepatitis. Nat. Commun..

[10] Ning BF (2010). Hepatocyte nuclear factor 4 alpha suppresses the development of hepatocellular carcinoma. Cancer Res..

[11] Lambert E (2020). Human hepatocyte nuclear factor 4-alpha encodes isoforms with distinct transcriptional functions. Mol. Cell Proteomics.

[12] Babeu JP, Boudreau F (2014). Hepatocyte nuclear factor 4-alpha involvement in liver and intestinal inflammatory networks. World J. Gastroenterol..

[13] Tanaka T (2006). Dysregulated expression of P1 and P2 promoter-driven hepatocyte nuclear factor-4alpha in the pathogenesis of human cancer. J. Pathol..

[14] Nowicki-Osuch K (2021). Molecular phenotyping reveals the identity of Barrett's esophagus and its malignant transition. Science.

[15] Tanaka M (2017). Revisions of international consensus Fukuoka guidelines for the management of IPMN of the pancreas. Pancreatology.

[16] Chellappa K (2016). Opposing roles of nuclear receptor HNF4alpha isoforms in colitis and colitis-associated colon cancer. Elife.

[17] Moffitt RA (2015). Virtual microdissection identifies distinct tumor- and stroma-specific subtypes of pancreatic ductal adenocarcinoma. Nat. Genet..

[18] Bailey P (2016). Genomic analyses identify molecular subtypes of pancreatic cancer. Nature.

[19] Collisson EA (2011). Subtypes of pancreatic ductal adenocarcinoma and their differing responses to therapy. Nat. Med..

[20] Birnbaum DJ, Finetti P, Birnbaum D, Mamessier E, Bertucci F (2017). Validation and comparison of the molecular classifications of pancreatic carcinomas. Mol Cancer.

[21] Camolotto SA (2021). Reciprocal regulation of pancreatic ductal adenocarcinoma growth and molecular subtype by HNF4alpha and SIX1/4. Gut.

[22] Kim J (2013). An iPSC line from human pancreatic ductal adenocarcinoma undergoes early to invasive stages of pancreatic cancer progression. Cell Rep..

[23] Cai SH, Lu SX, Liu LL, Zhang CZ, Yun JP (2017). Increased expression of hepatocyte nuclear factor 4 alpha transcribed by promoter 2 indicates a poor prognosis in hepatocellular carcinoma. Therap. Adv. Gastroenterol..

[24] Sang L (2022). The role of hepatocyte nuclear factor 4alpha (HNF4alpha) in tumorigenesis. Front. Oncol..

[25] Chellappa K (2012). Src tyrosine kinase phosphorylation of nuclear receptor HNF4alpha correlates with isoform-specific loss of HNF4alpha in human colon cancer. Proc. Natl. Acad. Sci. USA.

[26] Yang T (2021). Therapeutic HNF4A mRNA attenuates liver fibrosis in a preclinical model. J. Hepatol..

[27] Wang SC (2014). MicroRNA-122 triggers mesenchymal-epithelial transition and suppresses hepatocellular carcinoma cell motility and invasion by targeting RhoA. PLoS One.

[28] Kalimuthu SN (2020). Morphological classification of pancreatic ductal adenocarcinoma that predicts molecular subtypes and correlates with clinical outcome. Gut.

[29] Bankhead P (2017). QuPath: Open source software for digital pathology image analysis. Sci. Rep..

[30] Eeckhoute J (2003). Hepatocyte nuclear factor 4 alpha isoforms originated from the P1 promoter are expressed in human pancreatic beta-cells and exhibit stronger transcriptional potentials than P2 promoter-driven isoforms. Endocrinology.

[31] Dubois V, Staels B, Lefebvre P, Verzi MP, Eeckhoute J (2020). Control of cell identity by the nuclear receptor HNF4 in organ pathophysiology. Cells.

[32] Lv DD, Zhou LY, Tang H (2021). Hepatocyte nuclear factor 4alpha and cancer-related cell signaling pathways: a promising insight into cancer treatment. Exp. Mol. Med..

[33] Pan J (2020). Lineage-specific epigenomic and genomic activation of oncogene HNF4A promotes gastrointestinal adenocarcinomas. Cancer Res..

[34] Colleypriest BJ (2017). Hnf4alpha is a key gene that can generate columnar metaplasia in oesophageal epithelium. Differentiation.

[35] Jonckheere N (2012). GATA-4/-6 and HNF-1/-4 families of transcription factors control the transcriptional regulation of the murine Muc5ac mucin during stomach development and in epithelial cancer cells. Biochim. Biophys. Acta.

[36] Lussier CR, Babeu JP, Auclair BA, Perreault N, Boudreau F (2008). Hepatocyte nuclear factor-4alpha promotes differentiation of intestinal epithelial cells in a coculture system. Am. J. Physiol. Gastrointest. Liver Physiol..

[37] Amato E (2014). Targeted next-generation sequencing of cancer genes dissects the molecular profiles of intraductal papillary neoplasms of the pancreas. J. Pathol..

[38] Brunton H (2020). HNF4A and GATA6 loss reveals therapeutically actionable subtypes in pancreatic cancer. Cell Rep.

[39] Babeu JP, Jones C, Geha S, Carrier JC, Boudreau F (2018). P1 promoter-driven HNF4alpha isoforms are specifically repressed by beta-catenin signaling in colorectal cancer cells. J. Cell Sci..

[40] Noce V (2019). YAP integrates the regulatory Snail/HNF4alpha circuitry controlling epithelial/hepatocyte differentiation. Cell Death Dis..

[41] Yokoyama A (2011). Multiple post-translational modifications in hepatocyte nuclear factor 4alpha. Biochem. Biophys. Res. Commun..

[42] Schlesinger Y (2020). Single-cell transcriptomes of pancreatic preinvasive lesions and cancer reveal acinar metaplastic cells' heterogeneity. Nat. Commun..

[43] Ma Z (2022). Single-cell transcriptomics reveals a conserved metaplasia program in pancreatic injury. Gastroenterology..

[44] Peng J (2019). Single-cell RNA-seq highlights intra-tumoral heterogeneity and malignant progression in pancreatic ductal adenocarcinoma. Cell Res..

[45] Cui Zhou D, Jayasinghe RG, Chen S, Herndon JM, Iglesia MD, Navale P, Wendl MC, Caravan W, Sato K, Storrs E, Mo CK (2022). Spatially restricted drivers and transitional cell populations cooperate with the microenvironment in untreated and chemo-resistant pancreatic cancer. Nature Genetics..

[46] Han J, DePinho RA, Maitra A (2021). Single-cell RNA sequencing in pancreatic cancer. Nat. Rev. Gastroenterol. Hepatol..

