# Supplementary material for: Differential spatial distribution of HNF4α isoforms during dysplastic progression of intraductal papillary mucinous neoplasms of the pancreas
Source: Sci Rep. 2023 Nov 16;13:20088. doi: 10.1038/s41598-023-47238-x (PMC10654504; doi:10.1038/s41598-023-47238-x)
Supplement: Supplementary file 1 — Supplementary Legends. [file 41598_2023_47238_MOESM1_ESM.docx]

**SUPPLEMENTARY MATERIAL ONLINE**

**Supplemental Figure S1. Pan-promoter HNF4α nuclear staining grades for each component.**

a) Images represent the average pan-promoter HNF4α nuclear staining grade assigned for each component. The scale bar represents 100 µm. b) LG IPMN versus HG IPMN (bottom left hooked bar): p<0.0001; HG IPMN versus well-diff INV (bottom center hooked bar): p=0.0026; well-diff INV versus poorly diff INV (bottom right hooked bar): p<0.0001; LG IPMN versus poorly diff INV (dotted hooked bar): p=0.0384; HG IPMN versus poorly diff INV (dashed hooked bar): p=0.0002; Kruskal-Wallis of non-tumoral components (top left hookless bar): p<0.0001; Kruskal-Wallis of IPMN and invasive components (top right hookless bar): p<0.0001.

**Supplemental Figure S2. P1 HNF4α nuclear staining grades for each component.**

a) Images represent the average P1 HNF4α nuclear staining grade assigned for each component. The scale bar represents 100 µm. b) LG IPMN versus HG IPMN (bottom left hooked bar): p<0.0001; HG IPMN versus well-diff INV (bottom center hooked bar): p=0.8021; well-diff INV versus poorly diff INV (bottom right hooked bar): p=0.0066; LG IPMN versus poorly diff INV (dotted hooked bar): p=0.1808; HG IPMN versus poorly diff INV (dashed hooked bar): p=0.0087; Kruskal-Wallis of non-tumoral components (top left hookless bar): p<0.0001; Kruskal-Wallis of IPMN and invasive components (top right hookless bar): p<0.0001.

**Supplemental Figure S3. P2 HNF4α nuclear staining grades for each component.**

a) Images represent the average P2 HNF4α nuclear staining grade assigned for each component. The scale bar represents 100 µm. b) LG IPMN versus HG IPMN (bottom left hooked bar): p=0.0211; HG IPMN versus well-diff INV (bottom center hooked bar): p=0.6260; well-diff INV versus poorly diff INV (bottom right hooked bar): p=0.0001; LG IPMN versus poorly diff INV (dotted hooked bar): p<0.0001; HG IPMN versus poorly diff INV (dashed hooked bar): p<0.0001; Kruskal-Wallis of non-tumoral components (top left hookless bar): p<0.0001; Kruskal-Wallis of IPMN and invasive components (top right hookless bar): p<0.0001.
