# Supplementary figures and images for: Differential spatial distribution of HNF4α isoforms during dysplastic progression of intraductal papillary mucinous neoplasms of the pancreas
Source: Sci Rep. 2023 Nov 16;13:20088. doi: 10.1038/s41598-023-47238-x (PMC10654504; doi:10.1038/s41598-023-47238-x)

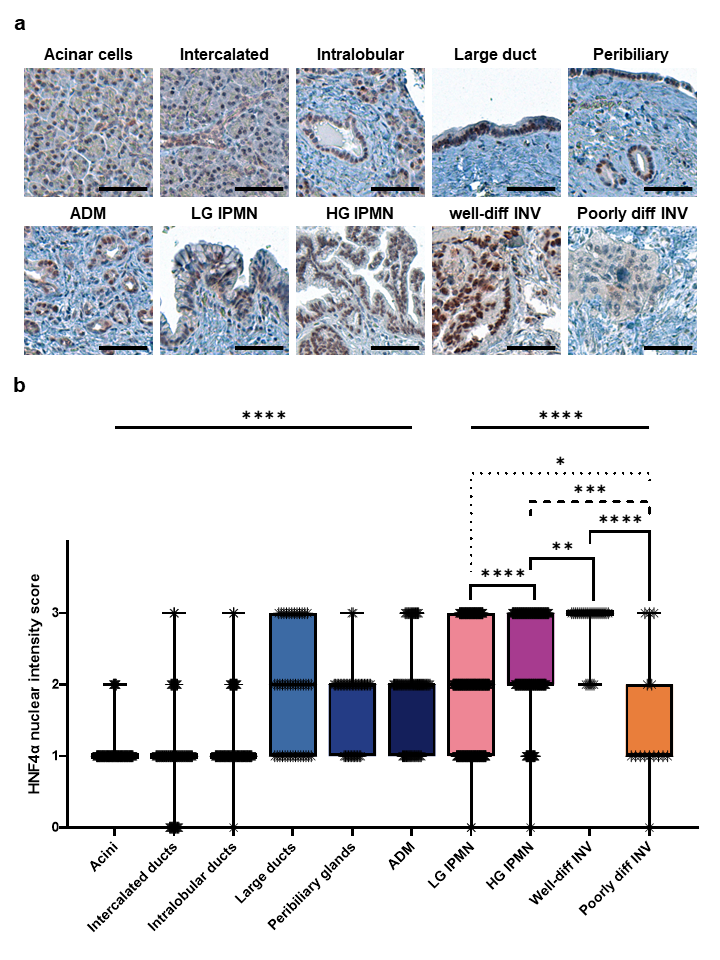

Supplement: Supplementary file 2 — Supplementary Figure S1. [file 41598_2023_47238_MOESM2_ESM.tif]

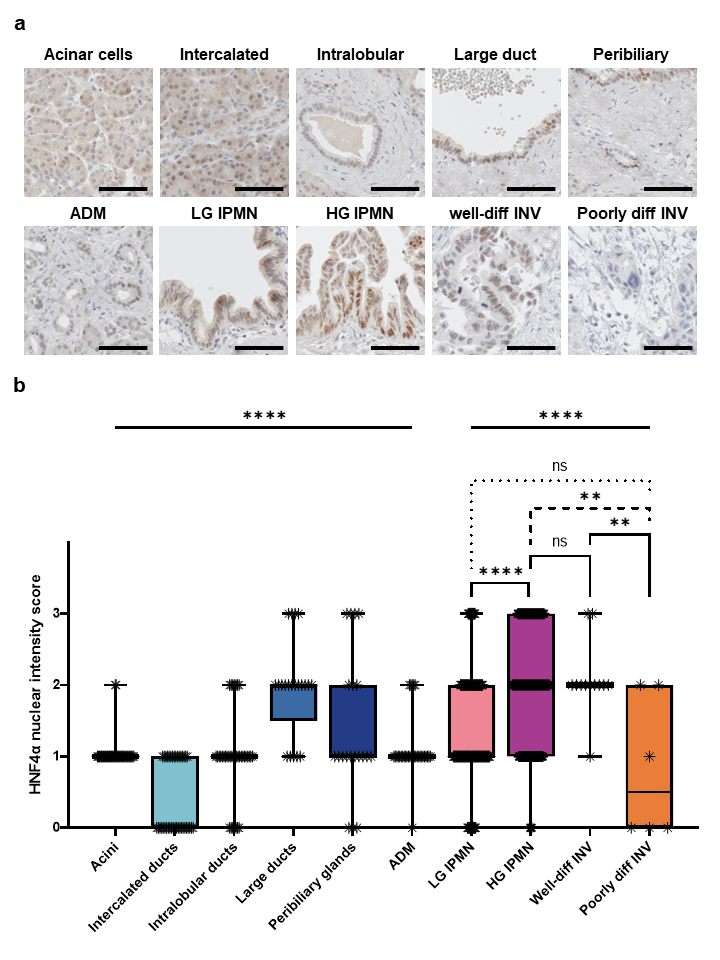

Supplement: Supplementary file 3 — Supplementary Figure S2. [file 41598_2023_47238_MOESM3_ESM.tif]

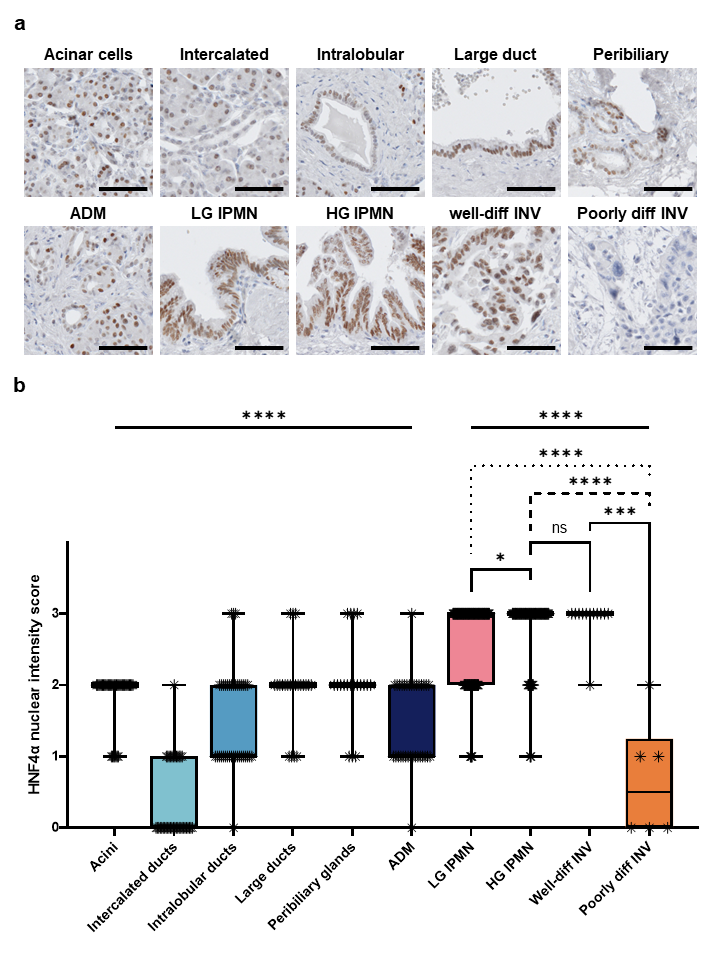

Supplement: Supplementary file 4 — Supplementary Figure S3. [file 41598_2023_47238_MOESM4_ESM.tif]
